# From detection to decision: how laboratory testing and reporting strategies can shape management of carbapenemase-producing *Enterobacterales* infections

**DOI:** 10.1128/asmcr.00139-25

**Published:** 2025-09-17

**Authors:** Shannon G. Murphy, Patricia J. Simner

**Affiliations:** 1 Department of Pathology, Johns Hopkins University School of Medicine 1500, Baltimore, Maryland, USA; 2 Mayo Clinic 6915, Rochester, Minnesota, USA; Rush University Medical Center, Chicago, Illinois, USA

## Abstract

Antimicrobial resistance mediated by carbapenemases significantly complicates treatment options. A case report by Morelan et al. (ASM Case Reports 1:e00094-25, 2025, https://doi.org/10.1128/asmcr.00094-25) illustrates how delayed recognition of *Klebsiella pneumoniae* carbapenemase (KPC) production in an *Escherichia coli* urinary tract infection resulted in suboptimal cefepime therapy, despite *in vitro* susceptibility. This case underscores the urgent need for clinical laboratories to implement on-site carbapenemase testing, as recommended by the Infectious Diseases Society of America and the Clinical and Laboratory Standards Institute (CLSI). Timely detection of KPC, coupled with reporting strategies for cefepime susceptibility results (suppression or editing results as resistant) in carbapenemase producers, may have facilitated earlier administration of a more appropriate antimicrobial and prevented recurrent infections and readmissions. In this commentary, we highlight recent updates to CLSI guidance for carbapenemase testing and antimicrobial susceptibility reporting strategies. Using this case report as an example, we outline the potential impact of these strategies on patient management and outcomes, further emphasizing the role of the clinical microbiology laboratory in antimicrobial stewardship.

## COMMENTARY

The rise of antimicrobial resistance mediated by carbapenemases significantly complicates treatment and poses an escalating threat to global health (1). Morelan et al. describe a case of urinary tract infection where delayed recognition of carbapenemase production led to the selection of suboptimal therapy (2). This case underscores recent calls from the Infectious Diseases Society of America (IDSA) and the Clinical and Laboratory Standards Institute (CLSI) to implement carbapenemase testing in clinical laboratories and adopt reporting strategies that help guide appropriate antimicrobial selection.

**Fig 1:**
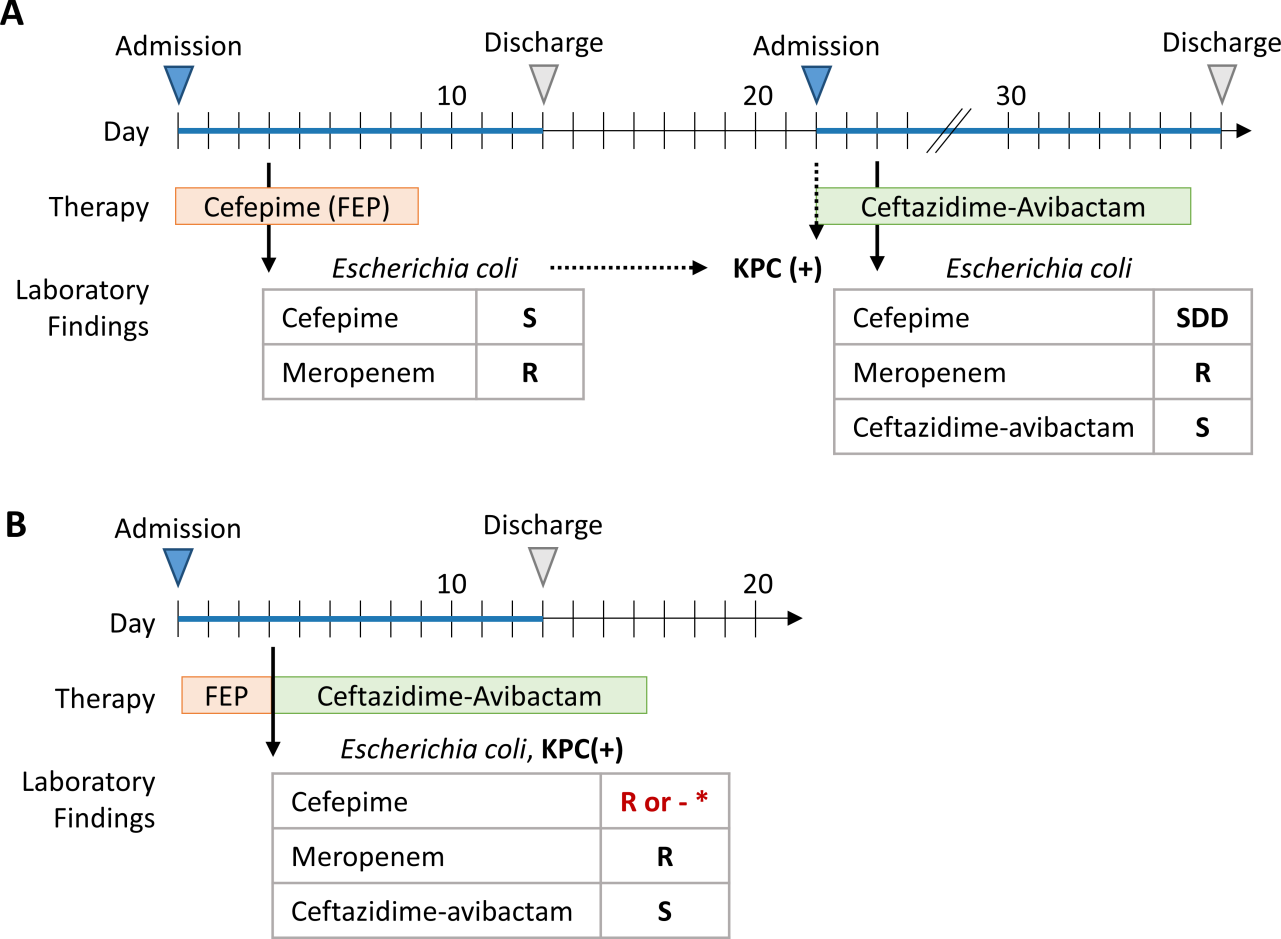
Potential impact of carbapenemase testing and updated antimicrobial reporting strategies on the course of a patient infected with a KPC-producing *Escherichia coli*. The timeline in panel **A** depicts the true course of a patient admitted with a urinary tract infection that failed cefepime therapy, as described in Morelan et al*. In vitro* susceptibility of the *E. coli* urine isolates to cefepime, meropenem, and ceftazidime-avibactam is indicated as susceptible (S), susceptible-dose dependent (SDD), resistant (R), or not released (-). The presence of carbapenemases (e.g., KPC+) is noted, if known. The duration of antimicrobial therapy with either cefepime (orange) or ceftazidime-avibactam (green) is shown. The timeline in panel **B** depicts the theoretical patient course if clinical laboratories implemented carbapenemase testing, modifed (or suppressed) cefepime in carbapenemase-producing *Enterobacterales* (*), and reported additional antimicrobial agents via a cascading algorithm.

This patient had a history of multidrug-resistant urinary tract infections and prior carbapenem exposure. A urine culture yielded >100,000 CFU/mL of *Escherichia coli* with *in vitro* susceptibility to cefepime (MIC, 1 µg/mL; susceptible) and resistance to meropenem (MIC ,>16 µg/mL) (Fig. 1A). The patient received a full course of cefepime and was discharged. Twenty-two days later, the patient was readmitted for recurrent infection with an *E. coli* that tested susceptible-dose dependent (SDD) to cefepime (MIC, 8 µg/mL). Review of state health laboratory testing revealed that the initial isolate, and presumably the isolate from the second admission, harbored a *Klebsiella pneumoniae* carbapenemase (KPC).

Despite testing susceptible (S) or SDD to cefepime, this agent is not recommended for the treatment of KPC-producing *Enterobacterales* (3). Studies have shown that although up to 37% of KPC-producing *Enterobacterales* test S or SDD to cefepime *in vitro* (4), this agent fails to demonstrate efficacy (i.e., a minimum 1-log reduction in organism) in *in vivo* murine models with human-simulated doses (5). Importantly, the Morelan et al. case report offers a clinical example of cefepime failure despite *in vitro* susceptibility. IDSA guidelines alternatively recommend the use of newer β-lactam-β-lactamase combination agents for the treatment of KPC-producing *Enterobacterales* (3), such as ceftazidime-avibactam or meropenem-vaborbactam. Following recognition that the patient’s infection was caused by a carbapenemase producer, the patient was successfully treated with ceftazidime-avibactam.

This case highlights two opportunities whereby clinical microbiology laboratories can help guide patient management. First, the carbapenemase testing status at the time of the initial infection was unknown, and IDSA (2024) and CLSI (2025) now encourage clinical laboratories to adopt routine carbapenemase testing (3, 6). Second, detection of resistance mechanisms can alter antimicrobial reporting strategies by suppressing or editing the result to resistant for inappropriate agents (e.g., cefepime) and reporting additional agents for consideration (i.e. cascade reporting). In the context of this clinical case, this commentary highlights how microbiology laboratories can utilize updated CLSI guidance for carbapenemase testing and reporting strategies to positively impact patient management and antimicrobial stewardship.

## DETECTION OF CARBAPENEMASE-PRODUCING ORGANISMS

Carbapenemase detection has historically been referred to public health entities that comprise the CDC Antimicrobial Resistance Laboratory Network, providing crucial national surveillance data (7). Given the shifting epidemiology of carbapenemase producers and the increased reliance on newer agents with mechanism-specific coverage, IDSA encourages clinical laboratories to implement methods for carbapenemase detection and differentiation to inform clinical care (3). In the case presented by Morelan et al., however, carbapenemase testing was not performed as part of standard care and State Health laboratory surveillance testing was not available until after patient discharge.

The criteria to screen for carbapenemases may vary based on several factors, including organism identification, local epidemiology, and national guidelines. Carbapenemases typically confer resistance to both cephalosporins and carbapenems, with exceptions (e.g., OXA-48, SME, IMI), and current screening recommendations include *Enterobacterales* that test resistant to one or more carbapenems (e.g., ertapenem, meropenem, imipenem) (6). An exception to this recommendation is *Proteus*, *Providencia*, and *Morganella* spp. that are only resistant to imipenem due to non-carbapenemase mechanisms (6). Ertapenem is generally the most sensitive (97%) marker for detection of the five most common carbapenemases in *Enterobacterales* (VIM, NDM, KPC, IMP, and OXA-48) (8). However, the specificity of ertapenem is relatively low (10–20%) due to the production of extended spectrum beta-lactamases (ESBLs) or AmpC beta-lactamases in combination with cell membrane-mediated mechanisms (e.g., porin mutations/efflux pumps). For this reason, CLSI notes that carbapenemase testing may not be necessary for *Enterobacter cloacae* complex and *Klebsiella aerogenes* (inducible AmpC beta-lactamase producers) that are only resistant to ertapenem, as resistance in these species is often due to non-carbapenemase mechanisms in the U.S (6). Importantly, these screening criteria are based on current CLSI breakpoints for carbapenems, with obsolete breakpoints (pre-2010) resulting in missed detections (9).

Methods for detecting and differentiating carbapenemases are described in CLSI M100 and in other reviews (6, 8). These algorithms may include phenotypic tests for broad carbapenemase production like the modified carbapenem inactivation method (mCIM) or Carba-NP. These methods can broadly differentiate resistance conferred by carbapenemase production versus non-carbapenemase-mediated mechanisms. Specific carbapenemases can be differentiated by molecular (gene-based) or lateral flow assay (LFA) (enzyme-based) tests, but these approaches are often limited to certain targets (i.e., VIM, NDM, KPC, IMP, and OXA-48). Testing algorithms may involve initial screening for carbapenemase production (e.g., mCIM) followed by a carbapenemase differentiation test (e.g., LFA), but workflows may be institution-specific and vary-based on prevalence in the patient population.

In the described case report, the *E. coli* isolate was identified as resistant to meropenem on hospital day four and subsequently referred to the state health laboratory for further characterization for surveillance purposes, with results reviewed on day 22 (Fig. 1A). Depending on the assays or algorithm used (e.g., rapid detection method, mCIM with overnight incubation), carbapenemase detection and KPC differentiation could have been provided on the same day as initial antimicrobial susceptibility results or the following day, respectively. This timely information could have facilitated a change in therapy from cefepime to an alternative agent (e.g., ceftazidime-avibactam) by hospital day 4 or 5 (Fig. 1B), potentially avoiding treatment failure and the need for patient readmission.

## INTERPRETATION AND REPORTING STRATEGIES

Antimicrobial test and report strategies are simple, effective, and cost-efficient methods that help guide appropriate antimicrobial selection (10). These institution-specific strategies should be developed in collaboration with antimicrobial stewardship programs and other stakeholders (11), but typically include both selective and cascade reporting (10). Selective reporting involves the release of only the agents that are most relevant to the organism and source of infection (e.g., release of fosfomycin for *E. coli* isolates from urine), whereas cascade reporting algorithms release additional agents based on the susceptibility profile of the organism. CLSI provides recommendations on test and report strategies in the recently updated Tables 1 in the M100 document; the suggested tiers outline agents for routine primary testing and reporting, as well as options for cascade reporting rules (6, 10).

In response to data showing reduced efficacy of cefepime for treating carbapenemase-producing *Enterobacterales*, updated CLSI documents recommend suppressing (or editing to resistant) cefepime S and SDD results (6). If routine carbapenemase detection and these reporting rules were in place for the described patient, the cefepime result would have been suppressed or changed to resistant, discouraging its use (Fig. 1B). Additionally, β-lactam-β-lactamase combination agents could be included in the cascade reporting rules for carbapenem-resistant or carbapenemase-producing *Enterobacterales* to be considered as a treatment option (3).

## CONCLUSIONS

The integration of timely carbapenemase detection and antimicrobial reporting strategies is key to informing optimal therapy for carbapenemase-producing *Enterobacterales*. In the case presented by Morelan et al., routine carbapenemase testing could have enabled earlier administration of appropriate therapy and possibly prevented recurrent infection and readmission. Laboratory algorithms and reporting strategies are informed by updated CLSI documents but should be tailored to reflect institution and population-specific needs and be developed collaboratively with other institutional stakeholders.
